# Academic-humanitarian partnerships: leveraging strengths to combat COVID-19

**DOI:** 10.1080/16549716.2020.1797296

**Published:** 2020-08-06

**Authors:** Adam R. Aluisio, Emily Zhu, Gabriela Gil, Thomas Kenyon, Vlatko Uzevski, Adam C Levine

**Affiliations:** a Department of Emergency Medicine, Brown University Warren Alpert Medical School, Providence, RI, USA; b Center for Human Rights and Humanitarian Studies, Brown University, Providence, RI, USA; c Brown University, Providence, RI, USA; d Project HOPE®, Millwood, VA, USA

**Keywords:** Coronavirus disease, COVID-19, pandemic, humanitarian response, training

## Abstract

The Coronavirus Disease 2019 (COVID-19) pandemic has overwhelmed many health systems globally. Innovative initiatives are needed to combat the pandemic and scaleup response efforts. This communication describes a collaborative partnership between an international humanitarian organization and an academic university to develop and rapidly deploy a remote digital COVID-19 trainer-of-trainers (TOT) program to enhance global response. The ongoing program has resulted in more than 900 TOT personnel who have themselves trained over 22,000 frontline response providers from 21 different countries and territories. The developed and implemented COVID-19 digital training program is a key example of how academic-humanitarian partnerships can be leveraged to strengthen healthcare training and response capacity during pandemics.

On 11 March 2020, the World Health Organization (WHO) declared the Coronavirus Disease 2019 (COVID-19) outbreak a pandemic [1]. Less than a month later it had progressed to over a million cases with infections identified in nearly every country globally [2]. Individuals infected with the severe acute respiratory syndrome coronavirus 2 (SARS-CoV-2) pathogen experience a range of states from asymptomatic to mild illness to severe disease requiring hospitalizations and intensive medical care. The drastic increase in the number of cases with limited treatment options and no proven vaccines has resulted in health systems becoming overwhelmed in high-, middle-, and low-income countries alike. These challenges in the pandemic are multifactorial, but driven largely by insufficient healthcare resources [3], and compounded by limited knowledge on the recently discovered causative virus.

Partnerships between academic institutions and humanitarian organizations are a unique and pragmatic approach to harness intersectoral collaboration and strengthen knowledge dissemination during pandemics. Previous emergencies have highlighted that such innovative partnerships have the potential to produce more sustainable and comprehensive public health responses [4]. While humanitarian organizations have established the logistical infrastructure and networks needed to efficiently and quickly address international emergencies, academic institutions house expertise in scientific understanding and educational programming [5]. Combining the complementary strengths of these two sectors facilitates a more practical repsonse approach by allowing personnel to function within there area of expertise while also being supported outside of that foci. Leveraging this interprofessional approach can result in a more efficient and robust response to global crises, such as the COVID-19 pandemic. Researchers and academic institutions are making progress in understanding the newly identified emerging infectious disease, SARS-CoV-2, and its associated disease states. Concurrently, humanitarian organizations around the globe are continuing to scale up their established partnerships with governments, healthcare facilities, and community-based organizations on the frontlines of the pandemic. These local partners are either preparing for, or currently treating, large numbers of individuals impacted and afflicted by COVID-19. To enhance global response, and leverage respective complementary expertise, Project HOPE®, an international humanitarian organization, partnered with Brown University’s Center for Human Rights and Humanitarian Studies (CHRHS) to develop a rapidly deployable trainer-of-trainers (TOT) program for responding to COVID-19.

The collaborative program developed by Project HOPE® and faculty from Brown University with specializations in infectious diseases, emergency care, humanitarian response, and education is designed to provide practical knowledge on preparing health systems and frontline providers for the COVID-19 pandemic. The training is grounded in a paradigm of interprofessional education, in which participants from two or more professional spheres (i.e. academics and humanitarians) engage to learn and share knowledge, resulting in trained providers competent in team-based healthcare delivery which can positively impact clinical outcomes [6–8]. The program teaches and evaluates participants on core competencies across eight focused modules including COVID-19 principles, infection prevention and control, screening and triage, diagnosis and management, stabilization and resuscitation, surge capacity, surveillance, and risk communication and community education. The content is based on vetted resources, from the WHO and other global advisory bodies as well as primary scientific research. Given the dynamic nature of the pandemic, the resources are updated as understanding evolves regarding best practices. The training employs interactive didactic modules, discussions, case-based learning, and video simulations to educate participants. The program has been delivered by Brown University educators to TOT participants from Project HOPE’s worldwide partners. With enrollment in and successful completion of the TOT program, participants have full access to the online digital materials to support scale-up in their local settings. The TOT program materials include teaching manuals, presentations, videos simulations, practicums, feedback forms and assessment questions to evaluate knowledge acquisition and competency among those secondarily trained by the TOTs. In order to expand access and utilization and better address the COVID-19 pandemic, Project HOPE® and Brown University have developed an open access version of the digital TOT program for global use (Supplement).

Thus far in implementation, the remote TOT program has been delivered to participants from 21 countries and territories, with over 900 personnel completing the program. The TOT participants have included a diverse cadre of healthcare actors including frontline clinical providers, support staff, public health personnel, local policymakers, and administrators. Future trainings are planned to further expand the reach to additional implementing partners globally (Figure 1). Although local scale-up is ongoing, augmentative dissemination has been successful with more than 22,000 frontline providers having been trained by the TOTs across the implementations settings thus far. Formal monitoring and evaluation of the TOT program are in process to further characterize dissemination and the programmatic impacts on local health systems, however some key lessons have been learned to address challenges. Theses exist in the domains of logistical coordination, technology access and management of content matter volume. Pertaining to coordination there was an early need identified to ensure that TOTs from each site were heterogenous in healthcare roles and affiliations, and to achieve this Project HOPE® personnel local to the training settings were tasked with purposefully recruiting diverse participants to enhance the network training cascades broadly. Given the remote approach delivered via online platforms in the program, some barriers to access were encountered particularly with bandwidth limitations. To address this, participants were able to use audio-only receipt of trainings, interactive text-based questions and answers periods during sessions and the training materials were provided for self-directed learning. Although SARS-CoV-2 is a novel emerging infectious disease the amount of available information on the pathogen is substantial and new research is reported at high rates since the discovery of the virus. Stemming from this, a challenge was recognized to ensure that the TOT program provides the most up to date information on COVID-19 from global advisory bodies while also delivering clear education around ongoing research outputs. To meet this challenge, the training materials have been continually updated as new guidance is released on COVID-19 and TOT sessions use a dual instructor approach in which the primary instructor focuses on the pre-set training material and a secondary instructor answers questions on the materials, related research and other inquires on COVID-19.

**Figure 1.**
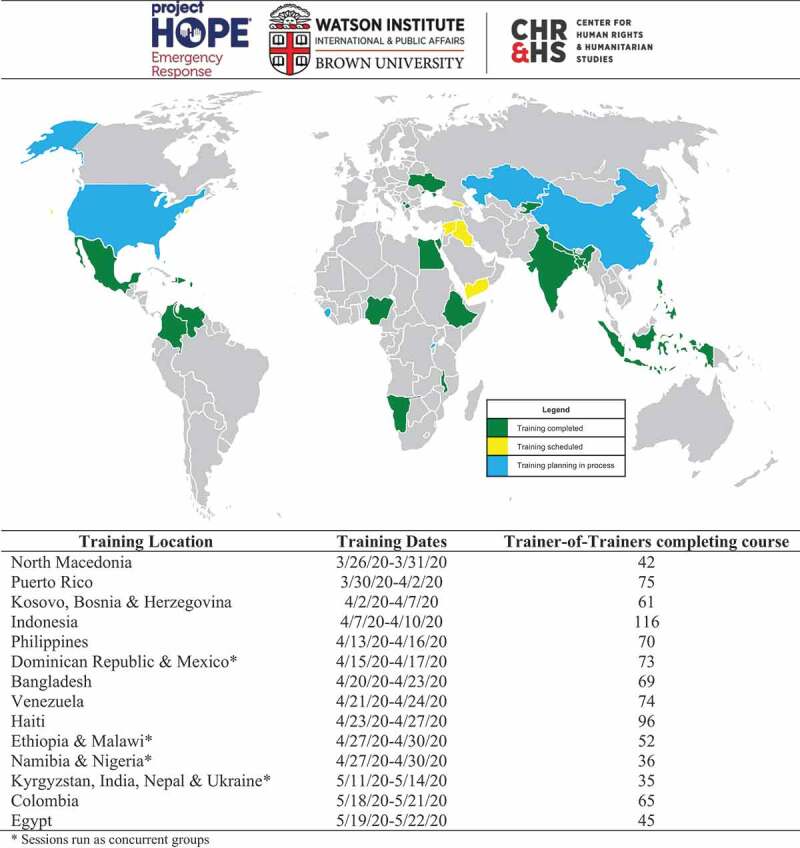
Global training implementation and dissemination.

There is a substantial need for innovative initiatives to address both current and future challenges faced by health systems as the world continues to respond to the COVID-19 pandemic. Collaborative academic-humanitarian programs represent a viable approach to strengthen the response to global healthcare crises. The developed and implemented COVID-19 digital training program is a key example of how academic-humanitarian partnerships can be leveraged to strengthen healthcare training and capacity during pandemics. Such partnerships can support enhanced dissemination and impacts by combining the logistical expertise and international networks of humanitarian actors with the educational resources from academic institutions. Moving forward, the Project HOPE®-Brown University CHRHS COVID-19 training program can serve as a model for the importance of the role of academic-humanitarian partnerships to augment healthcare response in pandemics and other global health emergencies.

## Paper in context

Barriers to pandemic responses exist and include limited trained frontline personnel. This communication outlines a collaborative partnership between an international humanitarian organization and an academic university to develop and rapidly deploy an online trainer-of-trainers program to enhance the global Coronavirus Disease 2019 pandemic response. Academic-humanitarian collaborations, which combine the logistical expertise of humanitarian actors with the educational resources from academic institutions are an impactful model that can be leveraged to strengthen global health response capacity.
